# Evaluation of anthropometric indices as a predictor of diabetes in Dong and Miao ethnicities in China: A cross-sectional analysis of China Multi-Ethnic Cohort Study

**DOI:** 10.1371/journal.pone.0265228

**Published:** 2022-03-11

**Authors:** Qianyuan Yang, Yalan Liu, Zhaofeng Jin, Leilei Liu, Zhiping Yuan, Degan Xu, Feng Hong

**Affiliations:** 1 Key Laboratory of Environmental Pollution Monitoring and Disease Control, School of Public Health, Guizhou Medical University, Ministry of Education, Guiyang, China; 2 University Town Hospital, Guizhou, China; 3 Guiyang City Center for Disease Control and Prevention, Guizhou, China; Polytechnic Institute of Coimbra: Instituto Politecnico de Coimbra, PORTUGAL

## Abstract

**Background:**

Although it is known that obesity is inseparable from diabetes, many anthropometric indices are used for determining obesity. At the same time, research on the predictive indices of diabetes in Chinese minority populations is lacking. Therefore, this study determines the relationship between different anthropometric indices and diabetes, and identifies the best index and best cut-off values for predicting diabetes.

**Method:**

In total, 11,035 Dong and Miao ethnic participants (age: 30–79 years) from the China Multi-Ethnic Cohort study were included. The logistic regression model was used to examine the relationship between the different anthropometric indices and diabetes risk. The receiver operating characteristic curve and the area under the curve (AUC) were used to identify the best predictor of diabetes.

**Results:**

In multivariate adjusted logistic regression models, body mass index (BMI), waist circumference (WC), waist-to-hip ratio (WHR), waist-to-height ratio (WHtR), a body shape index (ABSI), body roundness index (BRI), and visceral adiposity index (VAI) were positively correlated with diabetes risk. Among Chinese Dong men and women and Miao men, WHR had the largest AUC (0.654/0.719/0.651). Among Miao women, VAI had the largest AUC(0.701). The best cut-off values of WHR for Dong men and women and Miao men were 0.94, 0.92, and 0.91, respectively. The best cut-off value of VAI for Miao women was 2.20.

**Conclusion:**

Obesity indicators better predict diabetes in women than men. WHR may be the best predictor of diabetes risk in both sex of Dong ethnicity and Miao men, and VAI may be the best predictor of diabetes risk in Miao women.

## Introduction

Diabetes is a common metabolic disease characterised by chronic hyperglycaemia and is a serious threat to global health. As estimated by the International Diabetes Federation, approximately 463 million people had diabetes in 2019, and this number will reach 700 million by 2045 [1]. Traditional anthropometric indices are body mass index (BMI), waist circumference (WC), waist-to-hip ratio (WHR), and waist-to-height ratio (WHtR). Among them, BMI cannot distinguish between muscle tissue and fat accumulation [2]. The remaining three are indices of central obesity and are strongly correlated to abdominal fat [3]. Jamar et al. proved that WHtR is an independent predictor of insulin resistance, and its predictive effect is significantly greater than those of WC and BMI [4]. Another meta-analysis [5] and a cross-sectional analysis [6] have shown that WHtR is the best predictor of diabetes. However, some studies have also reported BMI or WC as the best predictor of diabetes [7–9]. New anthropometric indices such as a body shape index (ABSI), body adiposity index (BAI), visceral adiposity index (VAI), and body roundness index (BRI) have been recently proposed as alternative indices of obesity. ABSI can predict the risk of premature death independent of BMI [10]. In addition, ABSI is a sign of abdominal obesity and male insulin resistance [11]. BAI can accurately reflect body fat percentage [12]. VAI can not only distinguish between visceral adipose tissue (VAT) and subcutaneous adipose tissue (SAT) but is also related to insulin sensitivity [13]. Furthermore, abdominal fat accumulation varies with race, especially in the VAT [14]. Body fat mass and belly fat content are higher in women than in men [15].

China is a multi-ethnic country with 56 ethnic groups, and each ethnic group has different customs, living and eating habits. The Han people in China is the ethnic group with the largest population, accounting for 91.11% of the total population. In 1953, ethnic minorities accounted for 6.06% of the total population, and in 2020, ethnic minorities accounted for 8.89% of the total population, and their proportion showed an upward trend year by year. The Miao and Dong people in this study have a population of over one million, and are the main ethnic minorities in Qiandongnan Prefecture, Guizhou Province. "Global report on diabetes" pointed out that in 2014, the total prevalence of diabetes in China had reached 9.4%, and the prevalence rate of males was as high as 10.5%, and the proportion of deaths caused by diabetes accounted for 2%, ranked 7th, after cardiovascular disease, cancer, chronic respiratory disease, injury, infectious and maternal perinatal and nutritional disorders, and other non-communicable diseases. According to the World Health Organization, 39% of adults were overweight and 13% were obesity in 2016. In China, more than 30% of adults are obesity or overweight [16]. Only one study in China showed that Tibetans had a significantly lower prevalence of diabetes than Han Chinese, and little information was available about other ethnic groups [17]. It is well known that obesity contributes significantly to diabetes, however, this differs significantly by race/ethnicity and gender [18].

Because the body fat accumulation varies by race [14], the index that acts as the best predictor of diabetes in Dong and Miao populations in China remains unclear. Based on the China Multi-Ethnic Cohort (CMEC) study, we analysed the correlation between different anthropometric indices and diabetes in the Chinese Dong and Miao ethnic groups and determined the best cut-off values for the corresponding indices to provide evidence for diabetes prevention.

## Method

### Study population

**Fig 1:**
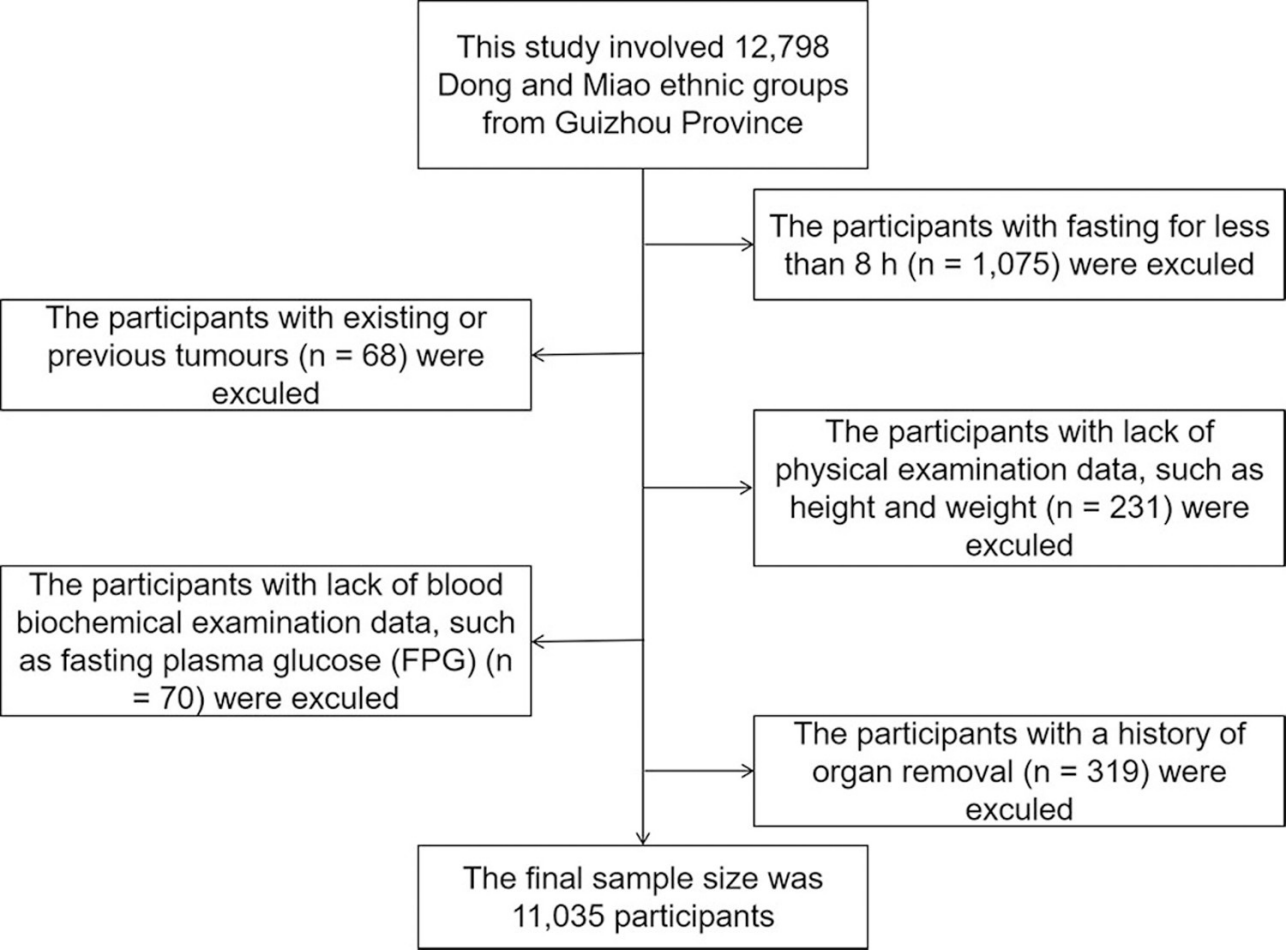
Flowchart showing the process of participants’ selection.

The present study participants were from the China Multi-Ethnic Cohort (CMEC) study, which has been recounted elsewhere [19]. From July 2018 to August 2019, a multi-stage, stratified cluster sampling method was used to obtain samples from the community population aged 30–79 years. In the first stage, the Qiandongnan Prefecture, China was selected as our study sites. In the second stage, four communities (Liping County, Congjiang County, Kaili City and Leishan County) in the settlement were selected by the local Centres for Disease Control and Prevention (CDCs), taking into account migration status, local health conditions and, most importantly, ethnic structure. In the final stage, all participants who met our inclusion criteria were invited to participate in our studies in consideration of both sex ratio and age ratio. Participants completed an epidemiological questionnaire including questions on socio-demographics, lifestyle, and medical history at baseline and underwent physical examination. The data of Dong and Miao population were obtained from the CMEC Study database. A total of 12.798 people from the Dong and Miao ethnic group were selected from the database, the participants with fasting for less than 8 h, existing or previous tumours, lack of physical examination data, lack of blood biochemical examination data, and a history of organ removal were excluded. The final study population involved 11,035 Dong and Miao ethnic groups from Guizhou Province (Fig 1). This study was approved by the Sichuan University Medical Ethical Review Board (K2016038) and the Research Ethics Committee of The Affiliated Hospital of Guizhou Medical University (2018[094]). Before data collection, all participants signed and submitted their informed consent forms.

### Data collection

First, a well-trained professional investigator conducted face-to-face interviews with the participants, which included questions about demographics, lifestyle, and medical history. Second, the fasting venous blood of the participants was collected by professional medical staff, and the samples were transported to Guizhou KingMed Diagnostics Group Co., Ltd for evaluating the levels of FPG, total cholesterol (TC), triglycerides (TG), high-density lipoprotein, and low-density lipoprotein (LDL-C) and glycated haemoglobin (HbA1c) by a fully automatic biochemical analyser (HITACHI 7180, Tokyo, Japan). Third, all survey participants underwent complete physical examination, which was conducted by trained medical professionals in accordance with standard procedures.

### Definition

An individual is considered to have diabetes in case of the following: fasting blood glucose ≥ 7.0 mmol/L; glycated haemoglobin ≥ 6.5%, and having diagnosed previously as having diabetes by a secondary hospital or above [20]. A person is considered a smoker if he/she has smoked more than 100 cigarettes so far [19]. A person is considered an ever smoker if he/she has quit smoking for more than half a year. BMI, WHR, WHtR, ABSI, BAI, BRI, and VAI were defined as follows [10, 12, 13, 21]:

$$BMI=\frac{wheight(kg)}{height{\left(m\right)}^{2}}$$ (1)

$$WHR=\frac{WC(\mathrm{cm})}{hip\_cirumference(cm)}$$ (2)

$$WHtR=\frac{WC(cm)}{height(cm)}$$ (3)

ABSI=WC(m)BMI23×height(m)12 (4)

$$BAI=\frac{\mathrm{hip}\_\mathrm{circumference}(\mathrm{cm})}{height{\left(m\right)}^{1.5}}-18$$ (5)

BRI=364.2−365.5×[1−(WC(m)2π0.5×height(cm))2]0.5 (6)

$$M\mathrm{ale}:\;VAI=\frac{WC\;(\mathrm{cm})}{39.68+1.88\times BMI}\times \frac{TG\;(\mathrm{mmol}/L)}{1.03}\times \frac{1.31}{HDL-C(mmol/L)}$$ (7)

$$Female:\;VAI=\frac{WC(cm)}{39.58+1.89\times BMI}\times \frac{TG(mmol/L)}{0.81}\times \frac{1.52}{HDL-C(mmol/L)}$$ (8)

### Statistical analysis

Continuous variables were described as mean ± SD, and categorical variables were described by frequency (percentage). The Mann–Whitney U test or chi-square test was used to evaluate the difference in indicators between different populations. After adjusting for age, smoking, drinking, educational level, hypertension, chronic hepatitis or hepatocirrhosis, coronary heart disease, hyperlipidaemia (except for VAI), and family history of diabetes, and grouping anthropometric indicators into tertiles, the logistic regression model was used to explore the association between anthropometric indices and diabetes, and its odds ratio (*OR*) and 95% confidence interval (*CI*) were calculated. The receiver operating characteristic (ROC) curve and the area under the curve (AUC) were used to identify the best predictor of diabetes. The inspection level was 0.05 (two-tailed). All statistical analyses were performed using IBM SPSS 25.0, Stata 15, and MedCalc 20.0.9 software.

## Results

### Demographic characteristics

Table 1 presents the basic demographic characteristics of all study participants by gender and ethnicity. The study included 11,035 participants (Dong men: 2,268; Dong women: 3,997; Miao men: 1,801; Miao women: 2,969), including 1,187 patients with diabetes (Dong men: 331, Dong women: 353, Miao men: 264, Miao women: 239). Compared with the Dong ethnic group, the average age, HBA1C, and LDL-C were lower in both sexes of the Miao ethnic group (*P* < 0.05), whereas DBP, BMI, HC, WHtR, TC, BRI, and BRI were higher in both sexes (*P* < 0.001). The number of current or ever smokers and drinkers was higher among the Miao women than among the Dong women (*P* < 0.05). SBP and WC were higher, whereas TG, FPG, and VAI were lower in the Miao women than in the Dong women (*P* < 0.05). WHR was lower in the Miao men than in the Dong men (*P* < 0.05).

**Table 1:** Demographic characteristics of study participants.

Variables	Dong ethnic		Miao ethnic	
	Male (2268)	Female (3997)	Male (1801)	Female (2969)
Age(years)	54.87±11.51	52.24±10.89	53.11±11.92^a^	50.97±11.48^b^
Education				
< high school	1657(73.06%)	3421(85.59%)	1232(68.41%)^a^	2451(82.55%)^b^
High school or vocational school graduate	255(11.24%)	245(6.13%)	240(13.32%)^a^	272(9.16%)^b^
≥College graduate	356(15.70%)	331(8.28)	329(18.27%)^a^	246(8.29%)^b^
Smoker				
Current smoker	1087(47.93%)	7(0.18%)	854(47.42%)	25(0.84%)^b^
Ever smoker	258(11.38%)	1(0.03%)	179(9.94%)	6(0.20%)^b^
Drinker	825(36.38%)	134(3.35%)	575(31.93%)^a^	212(7.14%)^b^
BMI (kg/m^2^)	23.87±3.46	23.83±3.49	24.79±3.28^a^	25.08±3.57^b^
WC (cm)	84.45±10.12	82.01±9.99	84.83±9.36	82.97±9.98^b^
HC (cm)	91.46±6.18	90.58±6.37	92.59±6.04^a^	92.06±6.48^b^
WHtR (cm/cm)	0.52±0.06	0.54±0.07	0.53±0.06^a^	0.55±0.07^b^
WHR (cm/cm)	0.92±0.07	0.90±0.08	0.91±0.07^a^	0.90±0.08^b^
VAI	2.45±3.68	2.57±4.21	2.27±2.87	2.23±3.15^b^
BRI	3.83±1.20	4.25±1.37	3.94±1.13^a^	4.47±1.44^b^
ABSI	0.08±0.004	0.08±0.01	0.08±0.004^a^	0.08±0.01^b^
BAI	26.36±3.14	30.60±3.73	27.37±3.17^a^	31.90±3.99^b^
SBP (mmHg)	129.40±18.55	121.78±18.95	130.50±19.00	123.44±20.21^b^
DBP (mmHg)	83.93±11.42	78.3610.67	84.90±11.57^a^	79.45±11.25^b^
Fasting blood glucose (mmol/L)	5.70±1.64	5.46±1.24	5.69±1.57	5.38±1.13^b^
HbA1c (%)	5.83±1.05	5.69±0.92	5.78±1.08^a^	5.63±0.83^b^
TC (mmol/L)	4.96±1.04	4.90±0.96	5.04±0.99^a^	4.99±0.97^b^
LDL-C (mmol/L)	2.96±0.90	2.97±0.85	2.89±0.83^a^	2.88±0.85^b^
HDL-C (mmol/L)	1.44±0.43	1.52±0.37	1.40±0.34	1.52±0.34
TG (mmol/L)	2.20±2.09	1.79±1.56	2.26±2.44	1.68±1.39^b^
the number of Hypertension	950(41.89%)	1111(27.80%)	806(44.75%)	849(28.60%)
the number of CHD	59(2.60%)	73(1.83%)	43(2.39%)	64(2.16%)
the number of chronic hepatitis or hepatocirrhosis	44(1.94%)	44(1.10%)	46(2.55%)	40(1.35%)
the number of Hyperlipidemia	1422(62.70%)	2142(53.69%)	1145(63.58%)	1496(50.39%)
the number of diabetes	331 (14.59%)	353 (8.83%)	264(14.67%)	239(8.05%)
Family history of diabetes	112(4.94%)	241(6.03%)	86(4.78%)	169(5.69%)

Data were expressed as mean ± SD continuous variables, and n (percentage) for categorical variables

^a^*P* < 0.001 compared with Dong ethnic men

^b^: *P* < 0.001 compared with Dong ethnic women.

The sample sizes were as follows: Dong ethnic men, n = 2,268; Dong ethnic women, n = 3,997; Miao ethnic men, n = 1,801; and Miao ethnic women, n = 2,969.

Abbreviations: BMI, body mass index; WC, waist circumference; WHtR, waist-to-height ratio; WHR, waist-to-hip ratio, ABSI, a body shape index; BAI, body adiposity index; BRI, body roundness index; VAI, visceral adiposity index; SBP, systolic blood pressure; DBP, diastolic blood pressure; CHD, coronary heart disease.

### Correlation analysis of anthropometric indices and diabetes

Table 2 presents the association between various anthropometric indices (namely BMI, WC, WHR, WHtR, ABSI, BAI, BRI, and VAI) and diabetes. After adjusting for age, smoking, drinking, education level, hypertension, chronic hepatitis or hepatocirrhosis, coronary heart disease, hyperlipidaemia (except for VAI), and family history of diabetes, the association between each index and diabetes were found to be significant (except for BAI). The *OR* values (95% *CI*) (Tertile 3 vs Tertile 1) of BMI, WC, WHR, WHtR, ABSI, BRI, and VAI among the Dong men were 2.08 (1.50, 2.90), 2.31 (1.61, 3.28), 2.51 (1.73, 3.66), 2.10 (1.50, 2.94), 1.56(1.11, 2.21), 2.14(1.52, 3.00), and 2.47 (1.82, 3.35), respectively, with VAI being the highest and ABSI being the lowest. The *OR* values (95% *CI*) (Tertile 3 vs Tertile 1) of BMI, WC, WHR, WHtR, ABSI, BRI, and VAI among the Miao men were 1.69 (1.13, 2.51), 2.63 (1.70, 4.08), 2.73 (1.78, 4.18), 2.66 (1.81, 3.92), 1.68 (1.19, 2.38), 2.69 (1.83, 3.97), and 2.63 (1.87,3.69), respectively, with WHR being the highest and ABSI being the lowest. The *OR* values (95% *CI*) (Tertile 3 vs Tertile 1) of BMI, WC, WHR, WHtR, ABSI, BRI, and VAI in the Dong women were 2.44 (1.81, 3.30), 4.17 (2.94, 5.90), 3.94 (2.78, 5.57), 3.64 (2.51,5.29), 2.41 (1.69, 3.45), 3.60 (2.48, 5.23), and 4.23 (2.92,6.12), respectively, with VAI being the highest and BMI and ABSI being the lowest. The *OR* values (95% *CI*) (Tertile 3 vs Tertile 1) of BMI, WC, WHR, WHtR, ABSI, BRI, and VAI in the Miao women were 1.86 (1.26, 2.75), 2.60 (1.71, 3.96), 2.65 (1.75, 4.01), 3.52 (2.07, 6.00), 1.84 (1.29, 2.64), 3.51 (2.06, 6.00), and 3.91 (2.60, 5.86), respectively, with VAI being the highest and ABSI being the lowest.

**Table 2:** Adjusted associations between anthropometric indices and diabetes.

Variables		Tertile 1	Tertile 2	Tertile 3	*P* for trend
		*OR*	*OR* (%95*CI*)	*OR* (%95*CI*)	
Dong/male	BMI	1(Reference)	1.49 (1.08,2.07)	2.08 (1.50,2.90)	<0.001
	WC	1(Reference)	1.19 (0.82,1.75)	2.31 (1.61,3.28)	<0.001
	WHR	1(Reference)	1.28 (0.86,1.89)	2.51 (1.73,3.66)	<0.001
	WHtR	1(Reference)	1.79 (1.30,2.46)	2.10 (1.50,2.94)	<0.001
	ABSI	1(Reference)	1.14 (0.81,1.61)	1.56 (1.11,2.21)	0.007
	BAI	1(Reference)	1.21 (0.92,1.60)	1.91 (1.27,2.87)	0.004
	BRI	1(Reference)	1.87 (1.35,2.57)	2.14 (1.52,3.00)	<0.001
	VAI	1(Reference)	1.68 (1.21,2.33)	2.47 (1.82,3.35)	0.028
Miao/male	BMI	1(Reference)	1.11 (0.74,1.67)	1.69 (1.13,2.51)	0.003
	WC	1(Reference)	1.81 (1.16,2.82)	2.63 (1.70,4.08)	<0.001
	WHR	1(Reference)	2.01 (1.31,3.08)	2.73 (1.78,4.18)	<0.001
	WHtR	1(Reference)	1.68 (1.15,2.44)	2.66 (1.81,3.92)	<0.001
	ABSI	1(Reference)	1.25 (0.90,1.74)	1.68 (1.19,2.38)	0.002
	BAI	1(Reference)	1.11 (0.82,1.49)	1.25 (0.82,1.89)	0.324
	BRI	1(Reference)	1.72 (1.18,2.50)	2.69 (1.83,3.97)	<0.001
	VAI	1(Reference)	1.37 (0.95,1.97)	2.63 (1.87,3.69)	0.003
Dong/female	BMI	1(Reference)	1.56 (1.15,2.13)	2.44 (1.81,3.30)	<0.001
	WC	1(Reference)	2.18 (1.52,3.12)	4.17 (2.94,5.90)	<0.001
	WHR	1(Reference)	1.75 (1.21,2.64)	3.94 (2.78,5.57)	<0.001
	WHtR	1(Reference)	2.04 (1.37,3.02)	3.64 (2.51,5.29)	<0.001
	ABSI	1(Reference)	1.70 (1.17,2.47)	2.41 (1.69,3.45)	0.007
	BAI	1(Reference)	1.02 (0.72,1.46)	1.18 (0.84,1.68)	0.185
	BRI	1(Reference)	2.04 (1.38,3.03)	3.60 (2.48,5.23)	<0.001
	VAI	1(Reference)	2.21 (1.49,3.26)	4.23 (2.92,6.12)	<0.001
Miao/female	BMI	1(Reference)	0.93 (0.60,1.44)	1.86 (1.26,2.75)	0.003
	WC	1(Reference)	1.70 (1.10,2.64)	2.60 (1.71,3.96)	<0.001
	WHR	1(Reference)	2.14 (1.40,3.28)	2.65 (1.75,4.01)	<0.001
	WHtR	1(Reference)	2.29 (1.31,4.00)	3.52 (2.07,6.00)	<0.001
	ABSI	1(Reference)	1.35 (0.91,2.00)	1.84 (1.29,2.64)	0.001
	BAI	1(Reference)	0.83 (0.48,1.42)	1.31 (0.80,2.14)	0.037
	BRI	1(Reference)	2.28 (1.31,4.00)	3.51 (2.06,6.00)	<0.001
	VAI	1(Reference)	1.36 (0.87,2.13)	3.91 (2.60,5.86)	<0.001

*P* for trend: Linear trend was tested using the median level of each quartile of adiposity measure.

Adjusted for age, smoking, drinking, educational level, hypertension, chronic hepatitis or hepatocirrhosis, coronary heart disease, hyperlipidaemia (except for VAI), and family history of diabetes.

### ROC analysis

**Fig 2:**
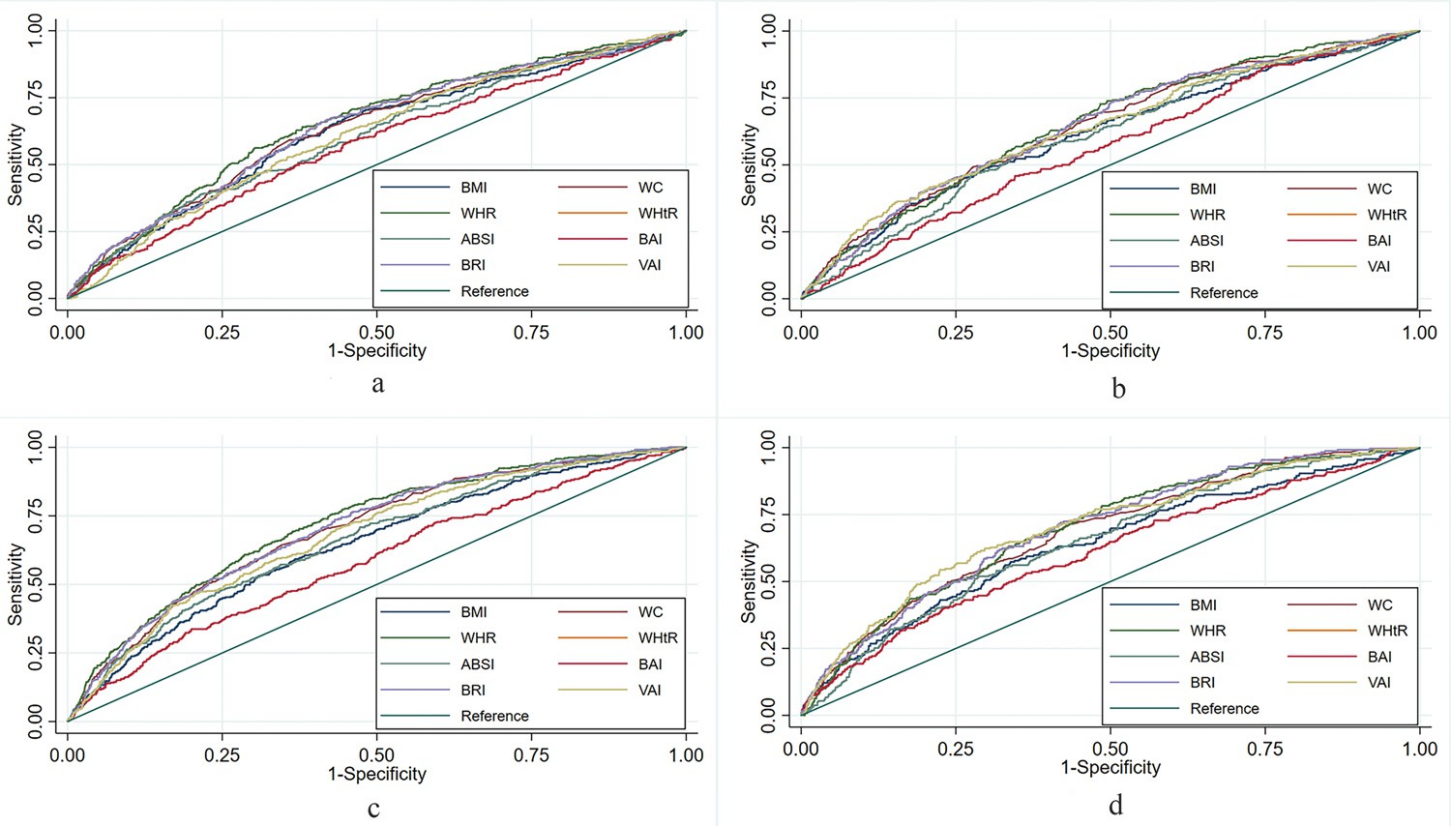
ROC (Receiver operator characteristic curves) for anthropometric indices to screen for diabetes in both sexes of the Dong and Miao ethnic groups. a is for the men of the Dong ethnic group, b is for the men of the Miao ethnic group, c is for the women of the Dong ethnic group, and d is for the women of the Miao ethnic group.

Fig 2 presents the ROC curves for the anthropometric indices of men and women in the Dong and Miao ethnic groups. Table 3 presents the best cut-off values of the eight anthropometric indices, which were determined based on the largest Youden index. Among Dong men, WHR, whose best cut-off value was 0.94, had the largest AUC (0.654, 95% *CI*: 0.634–0.674), and its predictive performance was significantly higher than that of BMI, ABSI, BAI, and VAI (*P* < 0.05), whereas WC, WHtR, and BRI had predictive capabilities similar to that of WHR (S1 Table). Among the Dong women, except for WHtR and BRI, and the predictive power of the other indices was weaker than that of WHR and the best cut-off value was 0.92 (*P* < 0.05). Among the Miao men, the predictive ability of ABSI and BAI was weaker than that of WHR and the best cut-off value of WHR was 0.91. Among the Miao women, the AUC of VAI was the largest (0.701, 95% *CI*: 0.685–0.718) and was significantly greater than that of BMI, ABSI, and BAI (*P* < 0.05), and the best cut-off value of VAI was 2.20.

**Table 3:** Ability of obesity factors to predict diabetes (AUC and 95% *CI*).

Variables	AUC	95%*CI*		cutoff	Sensitivity (%)	Specificity (%)
		Lower limit	Upper limit			
Dong/Male						
BMI	0.622	0.602	0.642	23.88	68.28	54.36
WC	0.636	0.616	0.656	87.9	59.21	64.12
WHR	0.654	0.634	0.674	0.94	64.05	62.05
WHtR	0.64	0.62	0.66	0.53	65.86	59.01
ABSI	0.607	0.586	0.627	0.08	38.97	78.42
BAI	0.576	0.555	0.596	26.41	58.91	54.31
BRI	0.640	0.62	0.66	3.98	65.86	59.01
VAI	0.611	0.591	0.631	1.58	61.93	55.55
Miao/Male						
BMI	0.617	0.594	0.639	26.36	47.35	71.89
WC	0.644	0.622	0.666	89.7	49.24	72.22
WHR	0.651	0.629	0.673	0.91	73.86	50.55
WHtR	0.648	0.625	0.67	0.52	73.48	49.97
ABSI	0.608	0.585	0.631	0.08	53.41	65.97
BAI	0.567	0.543	0.59	25.13	86.36	25.37
BRI	0.648	0.625	0.67	3.78	73.48	49.97
VAI	0.640	0.618	0.662	2.61	41.67	79.25
Dong/Female						
BMI	0.646	0.63	0.66	24.58	59.21	63.24
WC	0.7	0.685	0.714	85	64.31	65.5
WHR	0.719	0.705	0.733	0.92	69.41	63.57
WHtR	0.705	0.69	0.719	0.55	73.65	56.79
ABSI	0.662	0.647	0.676	0.08	70.82	52.79
BAI	0.586	0.57	0.601	33.28	33.71	79.96
BRI	0.705	0.69	0.719	4.3	73.65	56.79
VAI	0.68	0.666	0.695	1.89	70.25	56.57
Miao/Female						
BMI	0.640	0.622	0.657	26.14	57.32	66.15
WC	0.688	0.671	0.704	83.7	71.55	56.78
WHR	0.697	0.68	0.714	0.89	78.24	51.39
WHtR	0.699	0.683	0.716	0.56	71.97	58.06
ABSI	0.65	0.632	0.667	0.08	51.88	71.36
BAI	0.609	0.591	0.627	34.47	40.17	76.85
BRI	0.699	0.683	0.716	4.53	71.97	58.06
VAI	0.701	0.685	0.718	2.2	62.34	70.33

## Discussion

We herein studied the relationship between various anthropometric indices of the Dong and Miao ethnic groups of China and diabetes risk and compared new anthropometric indices with traditional anthropometric indices. Compared with other anthropometric indices (BMI, WC, ABSI, BAI, BRI, VAI, etc.), WHR is the best anthropometric index for predicting diabetes risk in Chinese Dong men and women, and Miao men because of its largest AUC, and its corresponding cut-off values were 0.94, 0.92, and 0.91, respectively. For Miao women, VAI, whose best cut-off value was 2.20, had the largest AUC. BAI was not suitable for predicting diabetes risk in the Chinese Dong and Miao people.

Luo et al. showed that preventing or delaying obesity or reducing cumulative exposure to obesity can significantly reduce diabetes risk [22]. BMI is the most commonly used index to define obesity. Hadaegh et al. found that BMI is the best predictor of the risk of type 2 diabetes mellitus (T2DM) in elderly people (age: <60 years) [23]. Steinbrecher et al. demonstrated that the AUC of BMI, which can better predict diabetes risk, is the largest among Caucasians, Native Hawaiians, and Japanese Americans, compared with those of WC, HC, WHR, and WHtR) [24]. Among Asians, BMI was also the most reliable index for predicting the risk of T2DM [25]. In addition, two cohort studies in China have found that BMI is the best predictor of diabetes risk in Chinese adults [7, 8]. However, Nevil et al. proposed that whether BMI can accurately represent obesity and can distinguish between obese and non-obese people remain to be investigated [2]. Subsequent studies have found that indices representing central obesity (WC and WHtR) predict fasting blood glucose, pre-diabetes, and diabetes better than BMI [3, 26, 27]. Hardy et al. revealed that WC is better than WHtR in predicting diabetes [9]. Skogberg showed that although WC and WHtR are the best indices for detecting diabetes, WHtR considers the height difference between individuals, and therefore is more reliable for diabetes risk assessment [28]. In China [6], Chile [29], and Jordan [30], studies have also shown that compared with WC and/or WHR, WHtR is the best anthropometric index for predicting diabetes. Our study revealed that compared with general obesity, central obesity (WC, WHR, and WHtR) has a better ability to distinguish diabetes from non-diabetes. Compared with non-diabetic patients, the abdominal fat of diabetic patients increases significantly [31]. Kang et al. also found that abdominal fat is significantly related to fasting blood glucose, insulin levels, and insulin resistance [15]. Through Mendelian randomisation analysis, Wang et al. found that abdominal obesity aggravates insulin resistance, leading to hyperglycaemia, which confirmed the potential causality between them [32].

Although traditional obesity indices such as WC can represent abdominal obesity, they cannot distinguish between VAT and SAT [13]. VAT has a stronger correlation with FPG than SAT [33]. Visceral obesity not only increases the risk of metabolic syndrome among adolescents but also increases the risk of cardiovascular disease in patients with diabetes [34, 35]. VAT is associated with diabetes in Chinese men and women, and its correlation is stronger than traditional anthropometric indices [36, 37]. At present, the gold standard for distinguishing between VAT and SAT is imaging technologies, such as computed tomography (CT) and magnetic resonance imaging (MRI), but due to their shortcomings such as high cost and inconvenient to use, they cannot be used for diabetes screening in a large population. In 2010, Amato proposed that VAI can be used in daily clinical practice and population studies to assess the cardiometabolic risk associated with visceral obesity [13]. In 2014, Chen et al. showed that compared with other measurement indices, VAI is a more accurate and more convenient surrogate index for VAT measurement and can be used to identify diabetes risk in large-scale epidemiological studies [38]. A study showed that VAI is an independent predictor of T2DM in adults in Qatar [39]. Many studies have proven that VAI is related to diabetes risk in the Chinese population, but it does not perform better than traditional indices such as BMI, WC, and WHtR [8, 40, 41]. Liu et al. showed that VAI has a better ability to predict diabetes [42]. In our study, VAI, which was the best index for predicting diabetes in the Miao women, had the largest AUC. Abdominal fat accumulation varies with race, especially in VAT [14]. Body fat mass and belly fat content are higher in women than in men [15]. Therefore, we believe that Miao women may have higher visceral fat than the other three subgroups, but indices such as WC cannot distinguish between SAT and VAT. In our study, compared with WHR, WC, and WHtR, VAI had relatively higher sensitivity and specificity. Therefore, compared with WC, WHR, and WHtR, VAI has a better ability to predict diabetes risk in Miao women.

In 2013, Thomas proposed a new anthropometric index, BRI, which is a predictor of body fat percentage and VAT [21]. Liu et al. also confirmed that BRI can be used as an effective index of visceral fat accumulation in patients with T2DM [43]. A study in northeastern China suggested that BRI can be used as a surrogate index for evaluating diabetes in the Chinese population [44]. Two other studies in China have also reported that BRI can be used as a surrogate index for predicting insulin resistance and diabetes [45, 46]. In this study, BRI was found to be a good predictor of diabetes risk, but it was not the best index. In 2012, Krakauer proposed that ABSI is significantly related to premature death in the general population [10]. Compared with BMI and WC, ABSI has a better ability to predict all-cause mortality, but it is not good at predicting chronic diseases [47]. In various studies, ABSI was not considered the best index for predicting diabetes risk [8, 48–50]. Including in this study, although ABSI is better than BMI, it is still not the best predictor of diabetes risk. In 2011, Bergman proposed that BAI has a strong correlation with DXA-derived obesity rates, which can be used to reflect the body fat percentage of adults of both sexes [12]. In our study, although BAI is an independent predictor of diabetes, its predictive power is far lower than that of the other indices, consistent with other studies [6].

In this study, the AUC of various anthropometric indices was greater for women than for men. After our stratification, the number of people in each group was reduced, especially in men, which may have led to the fact that obesity indicators did not show good predictive power in men because of the small sample size. In addition, obesity contributes significantly to diabetes, however, this differs significantly by race/ethnicity and gender [18]. As known, women have higher body fat than men. We therefore speculate that compared with obesity in men, obesity in women is more closely related to diabetes risk. Due to the difference in fat content between men and women and the distribution of fat between different ethnic groups, the best cut-off value for identifying diabetes is not exactly the same in different populations. In our ROC analysis, the ideal BMI thresholds for the Chinese Dong men and women and Miao men and women for identifying diabetes risk were 23.88, 24.58, 26.36, and 26.14, respectively, and the ideal WHR thresholds were 0.94, 0.92, 0.91, and 0.89, respectively. For the Chinese population, BMI ≥ 24.00 is defined as overweight and male WHR ≥ 0.9 and female WHR ≥ 0.8 are defined as central obesity. Xiao et al. reported WHR cut-off values of 0.92 and 0.85 for men and women from the Chinese Han population [6]. In our study, except for the Dong men, BMI cut-off values of the other subgroups were all higher than the national standard. Except among the Miao men, WHR cut-off values of the other subgroups were also significantly higher than the national standard and the Han ethnic group. Therefore, the same critical value should not be adopted between different ethnic groups and genders.

Our study has several limitations. First, this study only included baseline data and could not make causal inferences about the relationship between personal measurement indices and diabetes. Second, data on blood glucose after a 2-h oral glucose tolerance test were lacking, which may have led to the underestimation of diabetes prevalence. Our study also has some advantages. One advantage is that we are focusing on Dong and Miao ethnic groups in China, unlike previous studies that focused on the Han ethnic group. Moreover, our research used ethnic and gender stratification to obtain more targeted results and exclude the influence of gender.

## Conclusion

In conclusion, WHR may be the best anthropometric index for predicting diabetes risk. Its cut-off values in Chinese Dong men and women and Miao men were 0.94, 0.92, and 0.91, respectively. Miao women had higher visceral fat than the other three subgroups, and the index WC could not distinguish between SAT and VAT; thus, VAI could better predict diabetes risk in this population, with a cut-off value of 2.20, compared with the other indices. Although obesity is a risk factor for diabetes, it is a preventable and controllable factor. Our findings may contribute greatly to diabetes prevention.

## Supporting information

S1 Table P values for the AUC comparison of anthropometric indices. (XLSX)
